# Augmenting the Hospital Score with social risk factors to improve prediction for 30-day readmission following acute myocardial infarction

**DOI:** 10.18103/mra.v12i11.6089

**Published:** 2024-11

**Authors:** Iben Ricket, Michael E. Matheny, Ruth M. Reeves, Rashmee U. Shah, Christine A. Goodrich, Glenn Gobbel, Meagan E. Stabler, Amy M. Perkins, Freneka Minter, Chad Dorn, Bruce E. Bray, Lee Christensen, Ramkiran Gouripeddi, John Higgins, Wendy W. Chapman, Todd MacKenzie, Jeremiah R. Brown

**Affiliations:** 1 Departments of Epidemiology and Biomedical Data Science, Dartmouth Geisel School of Medicine, Hanover, NH; 2 Department of Biomedical Informatics, Vanderbilt University Medical Center, Nashville, TN; 3 Department of Biostatistics, Vanderbilt University Medical Center, Nashville, TN; 4 Division of General Internal Medicine, Vanderbilt University Medical Center, Nashville, TN; 5 Geriatric Research Education and Clinical Care Center, Tennessee Valley Healthcare System VA, Nashville, TN; 6 Division of Cardiovascular Medicine, University of Utah School of Medicine, Salt Lake City, Utah; 7 Department of Biomedical Informatics, University of Utah School of Medicine, Salt Lake City, Utah; 8 Utah Clinical & Translational Science Institute, University of Utah, Salt Lake City, Utah; 9 Centre for Digital Transformation of Health, University of Melbourne, Melbourne, Victoria, Australia

## Abstract

**Background:**

Hospital Score is a well-known and validated tool for predicting readmission risk among diverse patient populations. Integrating social risk factors using natural language processing with the Hospital Score may improve its ability to predict 30-day readmissions following an acute myocardial infarction.

**Methods:**

A retrospective cohort included patients hospitalized at Vanderbilt University Medical Center between January 1, 2007, and December 31, 2016, with a primary index diagnosis of acute myocardial infarction, who were discharged alive. To supplement ascertainment of 30-day readmissions, data were linked to Center for Medicare & Medicaid Services (CMS) administrative data. Clinical notes from the cohort were extracted, and a natural language processing model was deployed, counting mentions of eight social risk factors. A logistic regression prediction model was run using the Hospital Score composite, its component variables, and the natural language processing-derived social risk factors. ROC comparison analysis was performed.

**Results:**

The cohort included 6,165 unique patients, where 4,137 (67.1%) were male, 1,020 (16.5%) were Black or other people of color, the average age was 67 years (SD: 13), and the 30-day hospital readmission rate was 15.1% (N=934). The final test-set AUROCs were between 0.635 and 0.669. The model containing the Hospital Score component variables and the natural language processing-derived social risk factors obtained the highest AUROC.

**Discussion:**

Social risk factors extracted using natural language processing improved model performance when added to the Hospital Score composite. Clinicians and health systems should consider incorporating social risk factors when using the Hospital Score composite to evaluate risk for readmission among patients hospitalized for acute myocardial infarction.

## Introduction

Each year, approximately 635,000 adults in the United States (U.S.) have their first acute myocardial infarction (AMI)^1^. Nearly one in five adults hospitalized for an AMI will be re-hospitalized within 30-days of their incident discharge^1^. Hospital readmissions cost the U.S. healthcare system approximately 20 billion dollars annually^2^. Given the prevalence and high costs, hospital readmissions are a target for quality improvement and payment reforms^2,3^. As such, health systems are incentivized to identify patients at risk for hospital readmission proactively. Central to this effort are tools capable of enumerating risk for hospital readmission.

The Hospital Score is one predictive tool frequently used by clinicians to screen patients and identify those at greatest risk for hospital readmission^2,3^. The Hospital Score relies on eight clinical variables readily captured within most electronic health record (EHR) systems, including hemoglobin levels at discharge, discharge from oncology services, sodium level at discharge, procedure occurring during hospitalization, index admission type, number of hospital admissions in the prior year, length of stay and a flag indicating a length of stay longer than five days^2,3^. These eight variables, when combined, generate a composite score, which is widely used in a variety of medical specialties to enumerate risk for hospital readmission^2–6^.

The Hospital Score is a well-known and heavily researched readmission prediction tool^2,7,8^. Since the model underlying the Hospital Score is relatively simple and its candidate predictors are routinely collected among inpatient populations, it is easy to replicate in a variety of clinical settings^7^. For example, it was previously validated in the U.S., Canada, Israel, and Switzerland^8,9^. Compared to other predictive models, some studies found the Hospital Score to have superior discriminatory capabilities, despite its simplicity^2,3,9^. Other studies have achieved improved performance in predicting readmissions following AMI by leveraging robust modeling techniques or heterogenous EHR data^10–12^. While improved performance may be attainable with more robust modeling techniques, the Hospital Score offers the practical advantage of simplicity, transparency, and replicability while maintaining good performance^2^.

If the Hospital Score achieved modest performance improvements with slight modifications, it may offer an ideal tool for evaluating readmission risk. Modifying the Hospital Score by supplementing it with social risk factors may improve model performance, as these variables are known to affect risk for readmission^13^. Moreover, integrating social risk factors in models predicting hospital readmission previously demonstrated improvements in model performance^13–15^. The objective of this study was to test whether integrating social risk factors using a previously validated natural language processing (NLP) tool could improve the performance of the Hospital Score for predicting 30-day hospital readmission among patients with AMI.

## Methods

This study utilized a retrospective cohort of electronic health records (EHR) from patients attending Vanderbilt University Medical Center (VUMC). VUMC is a large, tertiary care facility in Nashville, Tennessee, serving a catchment area of nine surrounding states. The derivation of the retrospective cohort along with specific inclusion criteria are described elsewhere^11,16^. Briefly, patients hospitalized at VUMC with a primary diagnosis of AMI who were discharged alive between 1/1/2007 and 12/31/2016 were included in the cohort (N=6,165 unique patients). Patients who died before discharge were excluded (N=327). EHR data from the eligible cohort were harmonized to the Observational Medical Outcomes Partnership (OMOP) common data model (CDM). The OMOP CDM standardizes data and vocabularies for observational clinical data and is well utilized throughout the clinical research community^17–19^. The cohort was then linked to inpatient Medicare claims data to supplement ascertainment of 30-day readmissions.

This study made every reasonable attempt to adhere to the transparent reporting of multivariable prediction models for individual or diagnosis (TRIPOD) reporting standards (additional file 1)^20^. The VUMC institutional review board approved this study under expedited review with a waiver of informed consent. Consent was waived because the study was minimal risk with no patient interaction and could not be reasonably conducted if informed consent was required. Study staff followed all requisite provisions to ensure the privacy and confidentiality of data used in this study.

### DERIVING NATURAL LANGUAGE PROCESSING VARIABLES

Clinical notes were extracted for patients in the cohort using an NLP model called Moonstone. Moonstone is a rule-based NLP model previously validated on the VUMC cohort^16,21^. Methods related to Moonstone are well described in the literature^16,21–23^. Briefly, Moonstone was applied to a corpus of clinical notes from patients in the VUMC cohort. The corpus included clinical notes between the index AMI hospitalization and 30 days post-discharge. All notes were processed for eight measures of social risk, including: living alone, instrumental support, medication non-compliance (called medication compliance), impaired activity of daily life or impaired instrumental activities of daily life (ADL/IADL), a medical condition affecting ADL/IADL, dementia, depression, and language barrier^21^. Moonstone also determined whether each social risk factor was positive, negative, or uncertain (e.g., the text stated uncertainty about the patient’s depression). In addition, the attribute status of ‘any’ was generated, representing any positive, negative, or uncertain attribute status for each social risk factor^21^.

**Figure 1.**
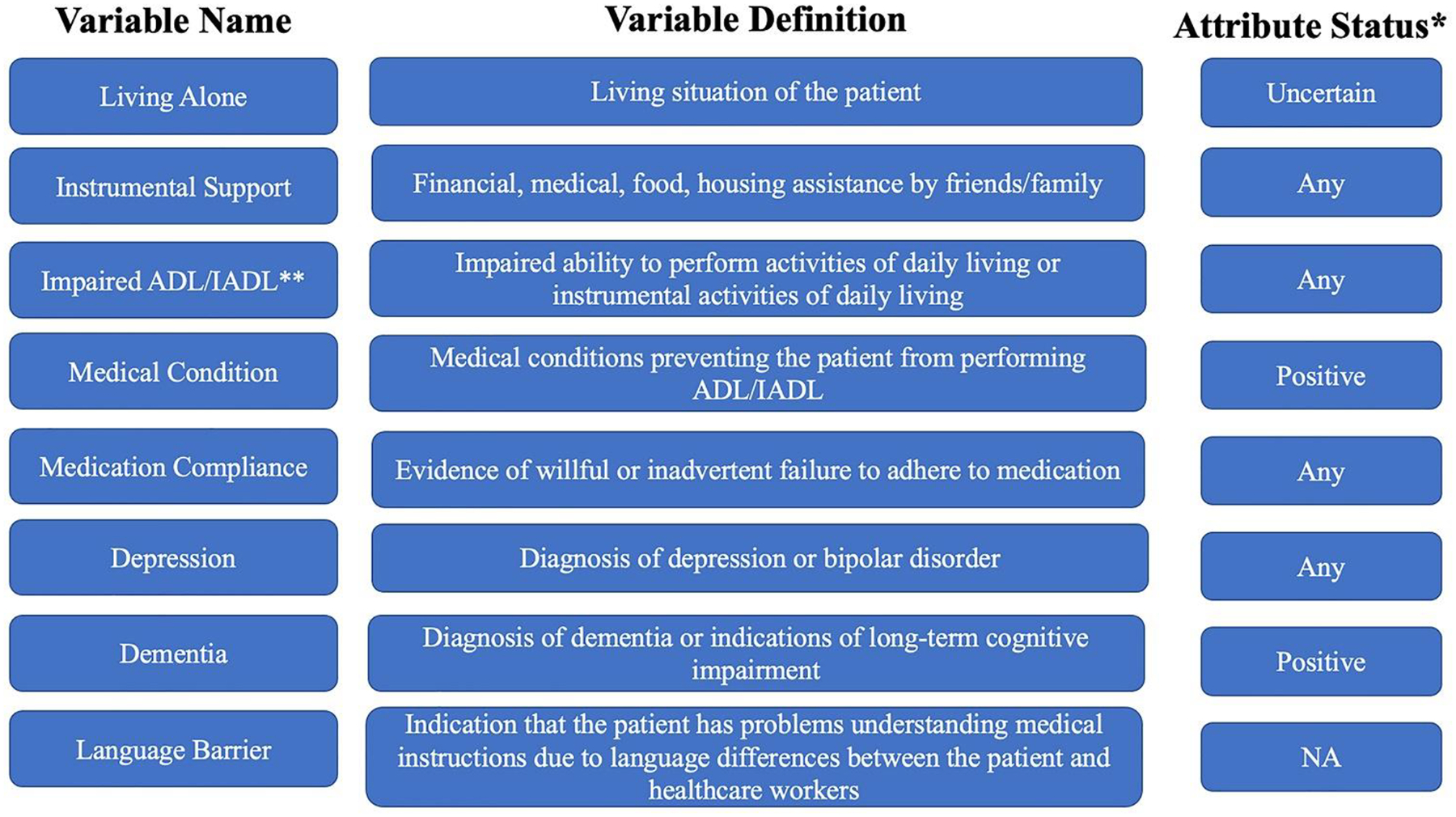
Definitions and attribute status for NLP-derived social risk factors^21^ *Attribute status included any, positive, negative, or uncertain **ADL: activities of daily living; IADL: instrumental activities of daily living Due to data completeness, language barrier was excluded from this study.

Once extracted by Moonstone, the eight social risk factor were rolled up to the encounter level^21^. This provided a binary indicator representing the presence or absence of each social risk factor and their associated attribution status (e.g., living alone positive expressed as 0 or 1). It was assumed that social risk factors were not present if the NLP system extracted none. Univariate analysis identified the most important attribute status for each of the eight NLP-derived social risk factors (NSRF), which were retained for subsequent analysis. Figure 1 contains information on each NSRF variable, including the attribute status used in this study. Due to issues of extreme missingness, the language barrier social risk factor was dropped from further analysis.

### HOSPITAL SCORE VARIABLE DEFINITIONS

Hospital Score was operationalized in two unique ways. The first definition used the Hospital Score composite, as defined in additional file 2. The second definition included the eight variables used in the composite score as individual candidate predictors, defined as Hospital Score component variables (additional file 2). Due to an artifact of the VUMC EHR system, the variable ‘procedure flag’ was present for the entire cohort. Since there was no variation in this variable for the entire cohort, it was not included in the analysis. As such, there were a total of seven Hospital Score component variables used in this analysis.

### OUTCOME

The primary outcome was all-cause 30-day hospital readmission. Readmissions included an observation or acute inpatient hospitalization within 30 days from the original AMI discharge (i.e., index). Readmissions excluded the following: rehabilitation admissions, nursing home admissions, or scheduled admissions for surgeries or procedures. Administrative databases from the included hospitals were used to derive the dates and causes of readmission. This included the admitting hospital state and surrounding state inpatient datasets and Medicare claims. This assured near-complete ascertainment of 30-day readmissions.

### ANALYTIC DATASET DEFINITIONS

This study generated five distinct data sets based on the Hospital Score variables and NSRF variables or some unique combination of them. Each of the five data sets described below included the outcome. The first dataset contained the Hospital Score composite variable (HS). The second dataset included the seven component variables used to define the Hospital Score (HSC). The third dataset included the seven NSRF variables (NSRF). The fourth dataset included the Hospital Score composite and the seven NSRF variables (HC + NSRF). Finally, the fifth dataset contained the seven component variables used to define the Hospital Score and the seven NSRF variables (HSC + NSRF). For clarity, the five unique dataset abbreviations are listed here: (1) HS, (2) HSC, (3) NSRF, (4) HS + NSRF and (5) HSC + NSRF, respectively.

### MISSING VALUES

To address missingness, 20 imputed data sets were generated using Markov chain Monte Carlo methods in SAS, assuming all imputed variables had a multivariate normal distribution^24^. All analysis (association & predictive) was executed separately on each of the 20 imputed data files. Corresponding results were pooled across the 20 imputed data files to generate a single value. Pooling of results followed Rubin’s rules, a comprehensive series of formulas and recommendations for averaging statistics and estimates across multiple files during implementation of multiple imputation^24^. All reported analysis described hereafter were run on each imputed file and results were pooled unless otherwise stipulated.

### UNIVARIATE, BIVARIATE & ADJUSTED ASSOCIATIONS

Prior to prediction model development, a series of descriptive statistics and basic association models were generated to enumerate unadjusted, bivariate, and adjusted associations between each variable and the outcome. Unadjusted associations were identified using univariate and bivariate logistic regression. To calculate adjusted associations, a logistic regression model with the seven component variables used to define the Hospital Score and the outcome was run. A second logistic regression model similarly evaluated adjusted associations between the seven NSRF variables and the outcome.

### PREDICTION MODEL DEVELOPMENT, ASSESSMENT & SCORING

Logistic regression prediction models were run on all five unique data sets previously described. Data were randomly split into training and testing sets using an 0.80/0.20 ratio, respectively. Each model was developed using 10-fold cross-validation on the training set with five repeats and performance was calculated using the complete hold-out test set. Model discrimination was assessed with pooled area under the receiver operator curve (AUROC) and 95% confidence intervals, both calculated on test sets^24^.

### MODEL COMPARISONS

Delong’s ROC comparison (ROC COMP) analysis was used to statistically compare performance of models built on the five unique data sets. Specifically, this occurred by statistically evaluating differences in the AUROCs obtained from these models. This included empirical comparisons between: (1) HS vs. HSC, (2) HS vs. NSRF, (3) HSC vs. NSRF, (4) HS vs. HS + NSRF, and (5) HSC vs. HSC + NSRF. From these analyses, the test statistics, standard error, and 95% confidence intervals were pooled across imputed files^24^. R 3.6.0 was used to conduct all statistical analysis along with model development, evaluation, and comparison.

## Results

Among 6,165 patients, 934 (15.1%) were readmitted within 30 days, 4,138 (67.1%) were male, 5,145 (83.5%) were white, and the average age was 65 years (SD=13 years). The study cohort included 1,938 (31.4%) with hypertension, 624 (10.1%) with coronary heart disease, and 502 (8.1%) with chronic kidney disease (additional file 3). Most of the study cohort (87.6%) were discharged to their homes (N=5,400). A more comprehensive table of patient characteristics is available in additional file 3.

### UNIVARIATE AND BIVARIATE NATURAL LANGUAGE PROCESSING-DERIVED SOCIAL RISK FACTOR VARIABLES

Moonstone was deployed on 93,670 clinical notes from patients in the cohort and included 46,123 total mentions of any social risk factor^16,21^. Instrumental support and impaired ADL/IADL were the two most prevalent NSRF variables across the study population (table 2). Among readmitted patients, 479 (51.3%) had records of instrumental support (status positive) compared to 1692 (32.2%) of patients without readmissions (table 2). Strong statistically significant associations between NSRF and the outcome were identified, including dementia (status positive) and living alone (status uncertain).

### UNIVARIATE AND BIVARIATE HOSPITAL SCORE VARIABLES

The average Hospital Score composite for the study cohort was 2.75 (SD=1.61). Almost half of the study cohort (N=2,788, 45.2%) were hospitalized for five or more days, and 240 (3.9%) had an oncology flag, indicating the presence of concurrent oncology care.

Patients with 30-day readmissions had larger values for the Hospital Score composite. Patients with longer hospital stays, those with records of hospital admissions in the prior year, and those with depressed hemoglobin (<12g/DL) prior to discharge were more likely to be readmitted (table 3).

### LOGISTIC REGRESSION PREDICTION MODELS ON FIVE UNIQUE DATA SETS

**Figure 2.**
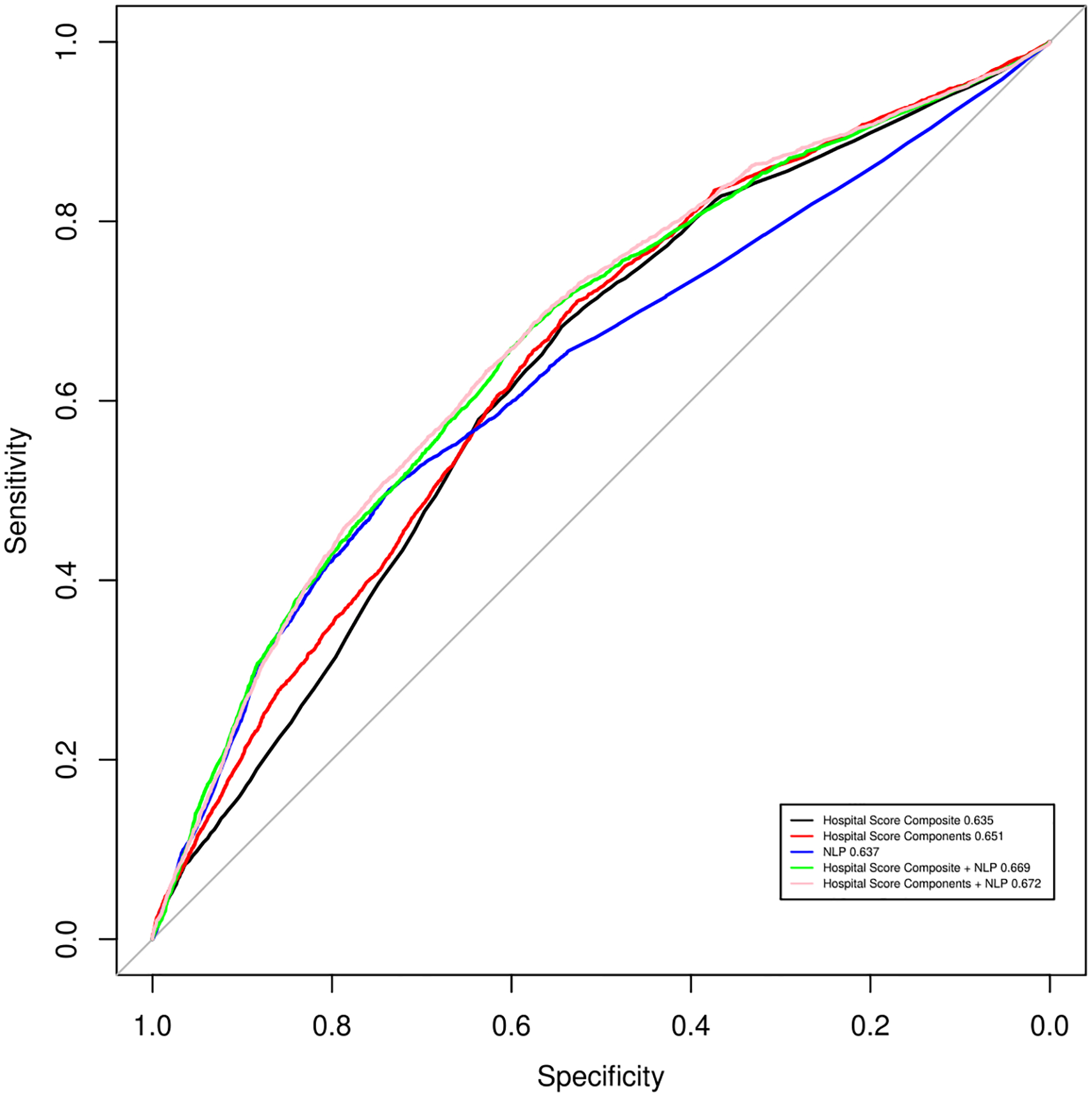
Pooled* area under the receiver operating curve (AUROC) from logistic regression prediction model using five unique combinations** of hospital score and NLP-derived social risk factor variables to predict 30-day hospital readmission following acute myocardial infarction among VUMC study cohort *Calculated on the test-set and pooled across 20 imputed files following Rubin’s rules **(1) HS, (2) HSC, (3) NSRF, (4) HS+NSRF, and (5) HSC + NSRF HS=Hospital Score Composite; HSC=Hospital Score component variables; NSRF=NLP-derived social risk factors

Table 4 contains pooled AUROC and standard errors from logistic regression prediction models deployed on five unique data sets. The data set containing HSC + NSRF obtained the highest AUROC (0.672), followed closely by the data set containing HS + NSRF (0.669). The data set with the lowest AUROC contained the HS (0.635), followed by the data set containing NSRF only (0.637). Figure 2 provides an illustration of pooled AUROC from all five unique data sets.

### COMPARING AREA UNDER THE RECEIVING OPERATING CURVE ACROSS FIVE UNIQUE DATA SETS

The Area Under the Receiving Operating Curve (AUROC) from models run on the five unique data sets were similar (table 5). The models run on HS and NSRF generated comparable AUROCs (0.635 vs. 0.637, respectively). Results from DeLong’s ROC COMP analysis identified 1 statistically significant difference between the AUROC from models using HS vs. HS + NSRF (pooled Z statistic: −2.951, pooled 95% CI: 0.005–0.062). No other statistically significant difference in AUROC was observed (Table 5).

## Discussion

Using prospective data from a large tertiary care facility, this study sought to evaluate the discriminatory performance of the Hospital Score, social risk factors, and their combination in predicting 30-day readmission following an AMI. Our study determined the model using HSC +NSRF obtained the best discriminatory performance. While this model achieved the highest AUROC, the inclusion of NSRF to the HSC variables did not statistically improve model performance. However, the inclusion of social risk factors to HS did generate a significant benefit to overall model performance. While the results are mixed, they demonstrate value in including social risk factors when using the HS for evaluating readmission risk among patients discharged following an AMI.

Prior research broadly supports using the Hospital Score before discharge to evaluate the risk for readmission among diverse patient populations^2,8^. For instance, in a multi-center validation study, the Hospital Score predicted 30-day readmissions with AUROCs between 0.68–0.78^8^. In a separate cohort of Taiwanese adults, the Hospital Score achieved an AUROC of 0.70. However, the authors improved prediction performance by generating another model using neural networks^25^. In addition to international validation, the Hospital Score has also been used to predict disease-specific readmissions, including heart failure^2,4^, AMI^2^, COPD^2^, pneumonia^2^, and neurosurgery^26^.

While the Hospital Score is primarily considered an efficient tool for evaluating readmission risk, recent research suggests expanding such predictive tools to include measures of social risk may improve model performance^15,27^. For example, prior research identified performance improvements with the inclusion of social risk factors in models predicting readmissions associated with percutaneous coronary interventions and heart failure^27,28^. However, other studies integrating social risk variables to supplement clinical prediction models has yielded mixed results. Most recently, Brown et al found no significant improvement in 30-day hospital readmission models with the integration of social risk factors derived from NLP^16^. Similarly, in 2 separate studies, Navathe et al and Wray et al found no improvement in models predicting readmissions when social risk factors were supplemented with clinical data^13,29^.

The mixed results of this study are worthy of comment, especially given the overwhelming evidence to support the association between social risk factors and outcomes in AMI^30,31^. The improvement in model performance seen when NSRF were added to the HS may reflect limitations in the composite algorithm to fully capture risk for readmission among our study population. In this case, the inclusion of NSRF to the HS may provide the model with more information needed to ascertain the risk for readmission. On the other hand, the lack of a statistically significant improvement in AUROC for models using HSC vs. HSC + NSRF could be a product of the NLP tool used in this study. Moonstone is run on clinical notes, which are known to contain variation between providers and contain bias, especially when compared to other more standardized measures (e.g., labs, vitals)^32,33^. Despite these limitations, NLP methods are well-researched tools for extracting social risk factors from clinical text and when added to the Hospital Score composite may offer clinicians an enhanced tool for evaluating risk of readmission among patients hospitalized with an AMI^13^.

## Limitations

Several limitations in this study require attention. First, this study utilized multiple imputation to address data missingness. While multiple imputation is considered robust, it may not easily be replicated at other sites or may need to be replaced with a less computationally intensive technique if used in production^34^. Second, patients under 65 years or those receiving Medicare fee-for-service did not have complete ascertainment of 30-day readmissions. Third, this study used logistic regression to predict 30-day readmissions, however, other algorithms may better characterize the data, which could lead to different results, including improved model performance. Fourth, the NSRF used in this study were limited to the presence or absence of seven constructs. However, many other important social risk factors have known associations with readmissions, including alcohol abuse, anxiety, or fall risk^13^. Fifth, the NLP-model Moonstone achieved good performance in extracting the seven NSRF used in this study (precision: 0.83 recall: 0.73 F1:0.78)^21^, however, its results were not perfect. Moreover, the assumption that no NLP extraction is equivalent to absence of the corresponding variable is imperfect. The absence of NLP-derived social risk factors may reflect true missing data (e.g., clinician not documenting the construct in the text) or a failure of the NLP model to extract the construct. Finally, the data used in this study are over ten years old, which may create limitations in the generalizability of our findings. Since the Hospital Score is internationally validated and the variables used in this study are routinely collected to this day, we believe this limitation is minimal.

## Conclusion

The Hospital Score remains an efficient tool for predicting risk of readmission among inpatient populations with a prior AMI. The value of social risk factors in supplementing the Hospital Score to estimate risk for 30-day readmission may depend on how the tool is used in practice (e.g., component variables vs. composite). Social risk factors may offer the greatest benefit when used to supplement the Hospital Score composite.

## Figures and Tables

**Table 1.** Abridged* characteristics of patients among VUMC study cohort

	Readmission (%) (N=934)	Non-readmission (%) (N=5231)
** Gender**		
Male	63.5 (N=593)	67.8 (N=3545)
Female	36.5 (N=341)	32.2 (N=1686)
** Race**		
White	83.7 (N=782)	83.4 (N=4363)
Black	10.8 (N=101)	9.4 (N=492)
Other	5.5 (N=51)	7.2 (N=376)
** Co-morbidities**		
Arrhythmia	21.0 (N=197)	12.7 (N=666)
Anemia	17.0 (N=160)	8.2 (N=430)
Hypertension	38.3 (N=358)	30.2 (N=1580)
COPD	4.5 (N=42)	2.9 (N=150)
CKD	16.0 (N=149)	6.7 (N=353)
Tobacco use	6.2 (N=58)	4.7 (N=246)
Depression	6.9 (N=64)	4.1 (N=217)
CAD	10.3 (N=96)	10.1 (N=528)
CHF	21.2 (N=198)	11.5 (N=599)
Dementia	2.6 (N=24)	1.9 (N=101)
Cardiac Arrest	5.7 (N=53)	5.1 (N=269)
STEMI	48.2 (N=450)	50.7 (N=2651)
Heart Failure during hospitalization	53.2 (N=497)	35.8 (N=1871)
Ischemia during hospitalization	17.0 (N=159)	11.5 (N=600)

Abbreviations: AMI=acute myocardial infarction; CAD=coronary artery disease; CHF=congestive heart failure; CKD=chronic kidney disease; COPD=chronic obstructive pulmonary disease; STEMI=ST-elevation myocardial infarction

* Full table available in additional file 3

**Table 2.** Pooled* univariate, bivariate, unadjusted, and adjusted associations of NLP-derived social risk factors and 30-day readmission following an acute myocardial infarction among VUMC study cohort

NLP-derived social risk factors	*Overall (%)*	*Non-Readmission N (%)*	*Readmissions N (%)*	*Unadjusted Odds Ratio*	*Adjusted Odds Ratio*
*Dementia positive*	171 (2.77)	114 (2.2)	57 (6.1)	2.920**	1.624**
*Depression any*	763 (12.38)	591 (11.3)	172 (18.4)	1.770**	1.322**
*Impaired ADL/IADL any*	1,679 (27.33)	1276 (24.4)	408 (43.7)	2.400**	1.164
*Instrumental Support any*	2,171 (35.21)	1692 (32.3)	479 (51.3)	2.200**	1.363**
*Living Alone uncertain*	893 (14.48)	626 (12.0)	267 (28.6)	2.940**	1.602**
*Medical Condition positive*	1,664 (26.99)	1257 (24.0)	407 (43.6)	2.440**	1.325**
*Medication Compliance any*	316 (5.13)	258 (4.9)	58 (6.2)	1.280	0.961

* Reported values were pooled across 20 imputed data files following Rubin’s rules

** significant at p<0.05

**Table 3.** Pooled* univariate, bivariate, unadjusted, and adjusted associations of hospital score variables and 30-day readmission following an acute myocardial infarction among VUMC study cohort

Hospital score variables	*Univariate*	*Outcome*	*Non-outcome*	*Unadjusted Odds Ratio*	*Adjusted Odds Ratio*
Continuous variables (mean (SD))					
Hospital Score Composite	2.75 (1.61)	3.42 (1.65)	2.63 (1.58)	1.338**	1.338**
LOS	5.94 (5.19)	7.47 (5.64)	5.67 (5.06)	1.056**	1.016
LOS5 Flag	2788 (45.2%)	588 (63.0)	2200 (42.1)	2.341**	1.721**
Prior Year Admissions Count	0.22 (0.74)	0.41 (1.21)	0.18 (0.61)	1.369**	1.317**
Categorical variables (count (%))					
Oncology Flag	240 (3.9)	57 (6.1)	183 (3.5)	1.793**	1.615**
Sodium Level Last 135 Flag	892 (14.5)	160 (17.1)	732 (14.0)	1.271**	0.993
Nonelective Admission Flag	77 (1.2)	15 (1.6)	62 (1.2)	1.361	0.930
Hemoglobin Level Last 12 Flag	2123 (50.7)	620 (66.4)	2503 (47.8)	2.152**	1.484**

* Reported values were pooled across 20 imputed data files following Rubin’s rules

** Significant at p<0.05

SD=standard deviation

**Table 4.** Pooled* area under the receiver operating curve (AUROC) and standard error (SE) from logistic regression models using five unique combinations of hospital score and NLP-derived social risk factor variables to predict 30-day hospital readmission following acute myocardial infarction among VUMC study cohort

Unique data sets	AUROC*	SE*
HS	0.635	0.029
HSC	0.651	0.033
NSRF	0.637	0.029
HS + NSRF	0.669	0.031
HSC + NSRF	0.672	0.033

* Calculated on the test-set and pooled across 20 imputed files following to Rubin’s rules

HS=Hospital Score Composite; HSC= Hospital Score component variables; NSRF=NLP-derived social risk factors

**Table 5.** Pooled* Z-statistic and p-value from ROC COMP analysis from logistic regression models predicting 30-day hospital readmission following acute myocardial infarction among VUMC study cohort

	HS vs. HSC	HS v. NSRF	HSC v. NSRF	HS vs. HS + NSRF	HSC vs. HSC + NSRF
Z statistic	−1.783	−0.069	0.672	−2.951	−1.852
AUC difference	0.016	0.002	−0.015	0.035	0.021
95% CI	−0.008, 0.038	−0.005, 0.056	−0.066, 0.038	0.005, 0.062*	−0.006, 0.048

* Calculated on the test-set and pooled across 20 imputed files following to Rubin’s rules HS=Hospital Score Composite; HSC= Hospital Score component variables; NSRF=NLP-derived social risk factors
